# Insights into the Evolution and Host Adaptation of the Monkeypox Virus from a Codon Usage Perspective: Focus on the Ongoing 2022 Outbreak

**DOI:** 10.3390/ijms241411524

**Published:** 2023-07-16

**Authors:** Jianglin Zhou, Xuejun Wang, Zhe Zhou, Shengqi Wang

**Affiliations:** Bioinformatics Center of AMMS, Beijing 100850, China; zjianglin@163.com (J.Z.); xjwang@bmi.ac.cn (X.W.)

**Keywords:** monkeypox virus, codon usage bias, mutation pressure, natural selection, evolution, host adaptation

## Abstract

The exceptionally widespread outbreak of human monkeypox, an emerging zoonosis caused by the *monkeypox virus* (MPXV), with more than 69,000 confirmed cases in 100 non-endemic countries since 2022, is a major public health concern. Codon usage patterns reflect genetic variation and adaptation to new hosts and ecological niches. However, detailed analyses of codon usage bias in MPXV based on large-scale genomic data, especially for strains responsible for the 2022 outbreak, are lacking. In this study, we analyzed codon usage in MPXV and its relationship with host adaptation. We confirmed the ongoing outbreak of MPXVs belonging to the West Africa (WA) lineage by principal component analysis based on their codon usage patterns. The 2022 outbreak strains had a relatively low codon usage bias. Codon usage of MPXVs was shaped by mutation and natural selection; however, different from past strains, codon usage in the 2022 outbreak strains was predominantly determined by mutation pressure. Additionally, as revealed by the codon adaptation index (CAI), relative codon deoptimization index (RCDI), and similarity index (SiD) analyses, the codon usage patterns of MPXVs were also affected by their hosts. In particular, the 2022 outbreak strains showed slightly but significantly greater adaptation to many primates, including humans, and were subjected to stronger selection pressure induced by hosts. Our results suggest that MPXVs contributing to the 2022 outbreak have unique evolutionary features, emphasizing the importance of sustained monitoring of their transmission and evolution.

## 1. Introduction

Human monkeypox is a zoonotic disease caused by the *monkeypox virus* (MPXV). The MPXV can be transmitted to healthy individuals through direct contact with infected animals, including monkeys, rodents, and other mammals [1]. The infection may also occur through respiratory droplets, close or direct contact with skin lesions, bodily fluids, contaminated fomites, and possible sexual contact [2]. Thereby, routine animal-to-animal transmission can lead to sporadic animal-to-human transmission, ultimately causing an outbreak or even epidemic as a result of further human-to-human transmission [3]. The MPXV is usually endemic in Africa, and sporadic infections outside of Africa are typically linked to a history of travel to endemic areas [4]. However, a multinational monkeypox outbreak began in 2022. Monkeypox was declared a Public Health Emergency of International Concern (PHEIC) by the World Health Organization (WHO). As of 4 October 2022, more than 69,000 laboratory-confirmed cases of monkeypox had been reported from 107 locations, including 100 locations that had not historically reported monkeypox [5]. Therefore, a detailed characterization of the MPXV genomes and their evolutionary dynamics is warranted.

MPXV belongs to the genus *Orthopoxvirus*, family *Poxviridae*, and is genetically closely related to the human pathogens *Variola virus* (VARV, causative agent of smallpox), *Cowpox virus* (CPXV), and *Vaccinia virus* (VACV) [6]. It is a large, double-stranded DNA virus. Its huge genome (≈197 kb) encodes about 190 nonoverlapping open reading frames. Although the natural reservoirs of MPXV are uncertain, MPXV infections have been documented in many host species, including humans, non-human primates, and many rodents [7]. Genetically, MPXV can be classified into two clades. The Central Africa (CA, or Congo Basin) clade is remarkably more virulent than the West Africa (WA) clade, with a mortality rate of 10.6% vs. 3.6% [1]. There is currently no MPXV-specific medication. The MPXV infections are generally self-limited and treated with supportive symptomatic therapeutics. Based on the genetic similarity of monkeypox and smallpox viruses, anti-smallpox virus drugs such as tecovirimat, cidofovir, and brincidofovir can be used for severe patients [3,8]. Additionally, two smallpox vaccines (JYNNEOS and ACAM2000) have been conditionally approved by the FDA for preventing MPXV infection, and the former showed real-world effectiveness in males [9]. Furthermore, several mRNA vaccines against MPXV have been developed and have displayed good safety and effectiveness in mice [10,11,12], providing the foundation for further clinical development of MPXV-specific vaccines.

Phylogenetic and codon usage analyses are used extensively to study the evolution of viruses. For instance, SARS-CoV-2 shows distinct codon usage characteristics and host adaptation from those of its ancestral Chiroptera-hosted coronaviruses [13,14,15]. The codon table is redundant; however, some codons are used more frequently than expected by chance, and this is referred to as codon usage bias (CUB). CUB in viruses results from complex interactions with hosts and is linked to many factors such as mutation pressure, natural selection or translation selection, and external environmental factors [16,17,18]. Analyses of CUB can provide valuable information about molecular evolution and host adaptation, hence deepening our understanding of the viruses and aiding in vaccine design. Although many traditional phylogenetic analyses of 2022 MPXV strains have been reported [19,20,21,22], a comprehensive study of CUB in MPXV, especially strains involved in the 2022 outbreak, has not been reported. Only one in silico study has reported the codon usage pattern of some MPXVs and concluded that mutation pressure plus selection at the codon level for optimal codon utilization, but not natural selection driven by hosts, contributed to the CUB of MPXV [23]. However, owing to the limitations of this previous work, including the small number of viral genomes (i.e., 13 strains), the focus on individual strains instead of clade-level patterns, the lack of similarity index analyses, and adaptation to hosts other than humans, a deeper comprehensive analysis is still needed. 

In this study, we comprehensively analyzed the codon usage pattern of the MPXV. The CUB of MPXV and its adaptation to multiple potential hosts were investigated. The findings of this study provide novel insights into the molecular evolution of MPXV, especially the strains responsible for the current outbreak.

## 2. Results

### 2.1. Phylogenetic Analysis of MPXV Strains of the 2022 Outbreak

**Figure 1:**
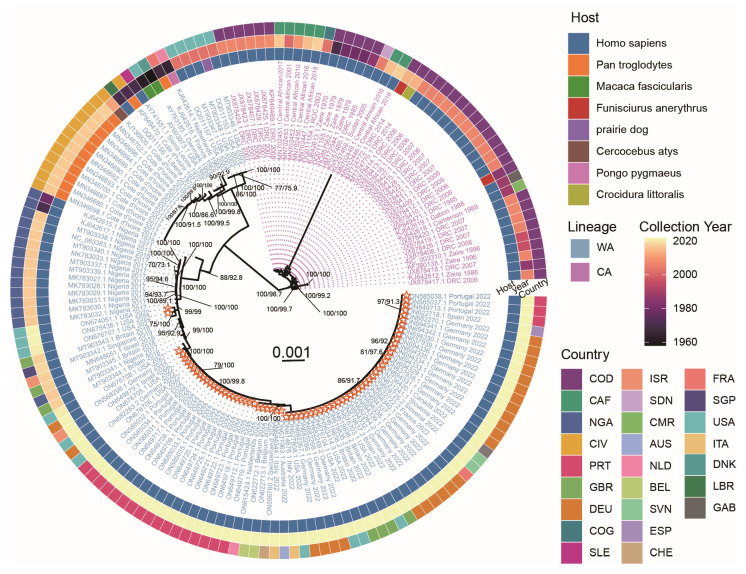
Maximum-likelihood phylogenetic tree of 161 MPXV strains based on full-length genomes. Viruses’ labels are filled according to their major lineage classification. The West Africa (WA) and Central Africa (CA) lineages are represented in blue and purple, respectively. Each 2022 outbreak strain is marked by an orange star. Numbers above the branches are ultrafast bootstrap support (%) and SH-aLRT support (%). For both metrics, only support values above 70% are displayed. The scale bar represents the expected substitutions per site. The circular color blocks at the periphery of the tree are distributed from inside to outside indicating the host, collection year, and country of the isolates, respectively. The country names are indicated using ISO alpha-3 codes.

To determine the phylogenetic relationships among the MPXV isolates, an ML phylogenetic tree was reconstructed based on whole genome sequences (Figure 1). Our results suggested that all 161 strains could be classified into two major lineages, corresponding to the previously reported CA and WA lineages, respectively. All viruses responsible for the 2022 worldwide outbreak belonged to the WA lineage. Despite their high similarity, these strains formed two distinct clades. Most of them (67/69) closely formed a monophyletic clade with an MPXV from a 2021 traveler from Nigeria to Maryland (ON676708), leading to the monkeypox epidemic in many countries of Europe and America. The other two strains from the United States (ON675438, and ON674051) clustered in another branch, with one strain isolated from a 2021 traveler from Nigeria to Texas (ON676707). These results suggested that the 2022 monkeypox outbreak may come from more than one origin.

### 2.2. MPXV Coding Sequences Are A-T Rich

**Figure 2:**
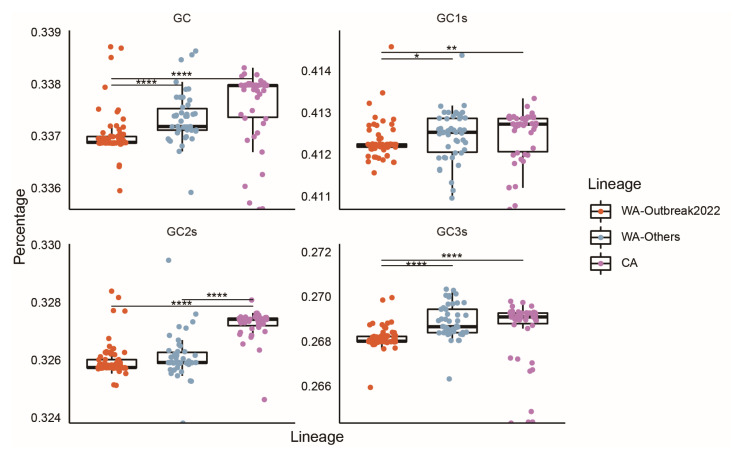
Boxplots of the GC, GC1s, GC2s, and GC3s values of the coding sequences of MPXV from different clades. The strains that caused 2022 worldwide monkeypox outbreaks are noted as “WA-Outbreak2022”. The isolates from previous outbreaks in the WA lineage are noted as “WA-Others”. Benjamini–Hochberg (BH)-corrected Dunn’s test was used for comparisons between groups. All differences with *p* < 0.05 are indicated. * *p* < 0.01; ** *p* < 0.01; **** *p* < 0.0001.

The nucleotide composition of MPXV was determined. The most abundant (%) mononucleotide was A (35.35 ± 0.03), followed by T (30.92 ± 0.04), G (18.08 ± 0.03), and C (15.65 ± 0.04). The prevalence (%) of nucleotides at the third codon position showed a similar pattern, i.e., abundance was highest for A3s (47.20 ± 0.06), followed by T3s (46.74 ± 0.07), G3s (19.22 ± 0.04), and C3s (17.80 ± 0.10). Additionally, the mean composition (%) of GC1s (41.24 ±0.05) was significantly higher than those of GC2s (32.64 ± 0.08) and GC3s (26.85 ± 0.10). The compositions (%) of GC (33.73 ± 0.06) and GC3s (26.85 ± 0.10) were lower than those of AT (66.27 ± 0.06) and AT3s (73.15 ± 0.10). Detailed nucleotide compositions of the MPXV strains are listed in Table S2. To precisely characterize the codon usage patterns of the coding sequences of MPXV, the strains responsible for the 2022 outbreak were further noted as WA-Outbreak2022, while the other strains in the WA lineage were noted as WA-Others. We found that the mean values of GC, GC1s, GC2s, and GC3s of WA-Outbreak2002 were significantly lower than those of the WA-Others and CA clades (Figure 2, adjusted *p* < 0.05, Dunn’s test). These data indicated that the coding sequences of MPXV are A- and T-rich, especially in WA-Outbreak2022.

### 2.3. CUB of the MPXV Coding Sequences

**Figure 3:**
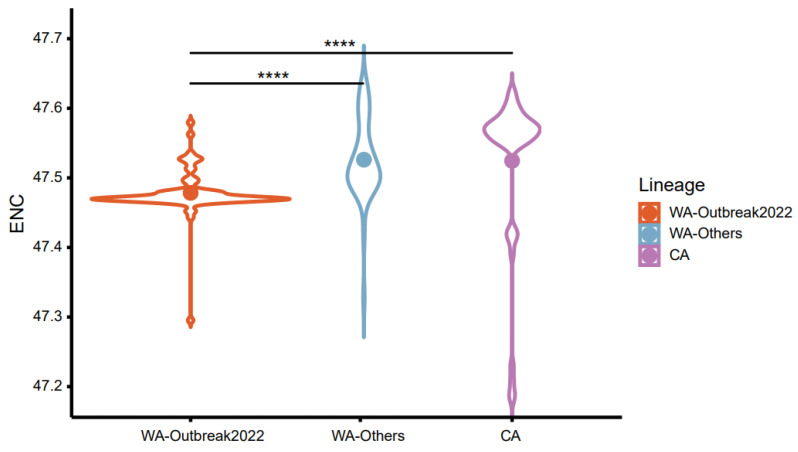
Violin plot of the ENC values of the MPXVs from different lineages. Benjamini–Hochberg (BH)-corrected Dunn’s test was performed to infer the significance of differences between groups. All differences with *p* < 0.05 are indicated. **** *p* < 0.0001.

To evaluate the degree of the MPXV CUB, the ENC values were estimated. The overall ENC values of MPXV strains were 47.51 ± 0.07, suggesting that the CUB of the MPXV isolates was relatively weak. Considering clade classification, the average ENC values of WA-Outbreak2022 (47.48 ± 0.03) were slightly but significantly lower than those of WA-Others (47.53 ± 0.06) and CA (47.52 ± 0.11) (adjusted *p*-values < 0.0001, Dunn’s test, Figure 3), indicating that CUB was slightly elevated in the 2022 outbreak strains.

### 2.4. Relative Synonymous Codon Usage (RSCU) of MPXV

To explore the synonymous codon usage preferences in the MPXV, the RSCU analysis was performed. The codons were used as expected by the literature [24] (Table S3). A total of 28 of 59 codons were preferred codons (RSCU > 1.0). The most preferred codons (27/28) were A/T-ended (14 T-ended, 13 A-ended) and only one codon (TTG) was G-ended. Out of 59, 4 synonymous codons (TTA, TCT, AGA, and GGA) were over-represented (RSCU > 1.6) and all of these were A/T-ended. A total of 23 codons were under-represented and all of these were G/C-ended (14 C-ended and 9 G-ended) (Table S3). These results suggested that A/T-ended codons are preferred in the coding sequences of the MPXV. Interestingly, although there were differences in abundance, the preferred, over- and under-represented codons were shared in all three clades.

Furthermore, the RSCU values of MPXV were compared with those of their potential hosts (Table S3). Two codons (ATT and CCT) were preferred in MPXV and all of its hosts, and only one codon (TCG) was under-represented in MPXV and all of its hosts. Individually, the commonly preferred codons between MPXV and its hosts ranged from 8 to 13, while commonly unpreferred codons (RSCU < 1.0) ranged from 12 to 18, regardless of the clade. These results indicated a mixture of coincident and antagonistic codon usage preferences between the MPXV and its hosts. 

### 2.5. Trends in Codon Usage Variation in MPXV Clades

**Figure 4:**
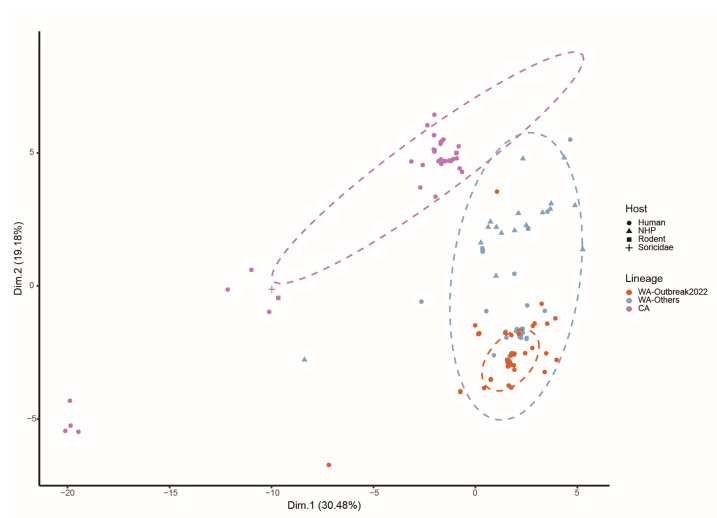
PCA of MPXV based on RSCU values for 59 synonymous codons. The ellipses show the 95% confidence interval. Each dot represents an MPXV strain. WA-Outbreak2022, WA-Others, and CA clades are depicted in orange, blue, and purple, respectively. The shape of the dot represents the host categories: human (circle), non-human primates (NHP, triangle), rodents (rectangular), and Soricidae (crisscross).

To explore the variation in codon usage among the coding sequences of MPXV, a PCA analysis was conducted. The first two principal components (Dim.1 and Dim.2) accounted for 30.48% and 19.18% of the total variance, respectively (Figure 4). According to the PCA plot, all strains clustered into two separate groups, corresponding to two major clades, WA and CA. In addition, despite clustering in a relatively closed subgroup, the variants isolated from the 2022 worldwide outbreak were still within the 95% confidence ellipse generated by the WA-Others strains, consistent with the results of the phylogenetic analysis. 

### 2.6. Mutation Pressure and Natural Selection Jointly Shaped Codon Usage Patterns of MPXV

**Figure 5:**
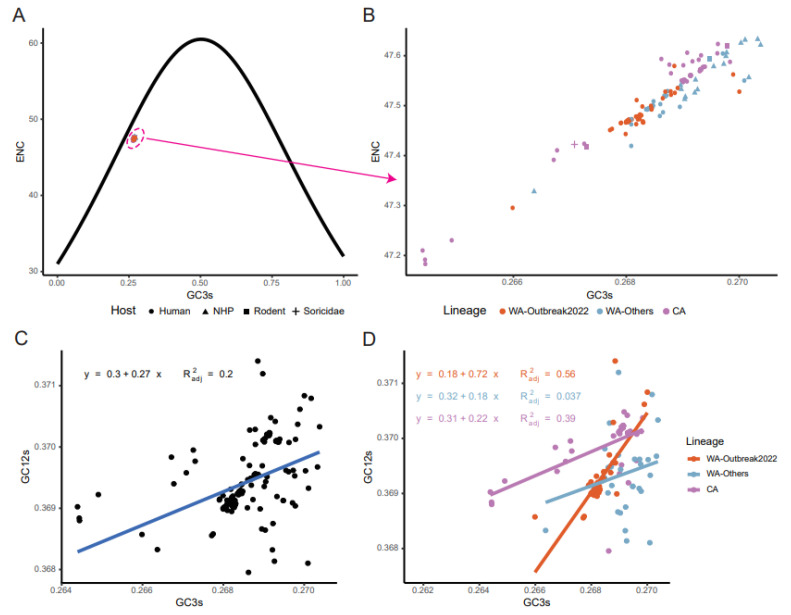
ENC-plot and neutrality analysis. ENC-plot of the coding sequences of the MPXV (**A**) and partially enlarged view (**B**). Neutrality plot analysis of all MPXV strains (**C**) and individual lineages (**D**). The continuous black line represents the expected ENC values. The interpretation of the color and shape are the same as described in Figure 4.

**Figure 6:**
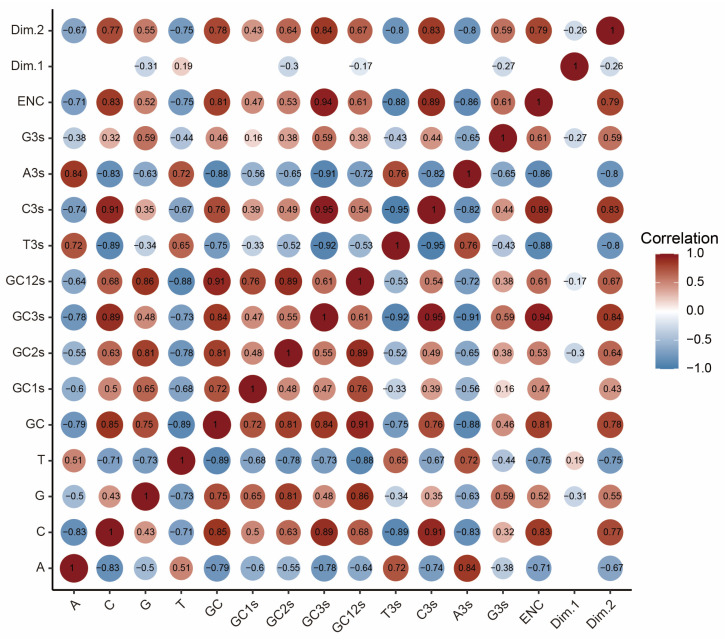
Spearman’s correlation analysis of the nucleotide composition, ENC, and the first two principal components (Dim.1 and Dim.2) of the PCA for MPXV coding sequences. Dark red and blue indicate a positive and negative correlation, respectively. Deeper colors indicate a higher correlation. Only significant correlations are displayed (*p* < 0.05).

To clarify the potential roles of mutation pressure and natural selection in driving the codon usage pattern of MPXV, an ENC-GC3s plot, correlation analysis, and neutrality analysis were performed. The ENC-GC3s plot revealed that all strains were near but below the expected curve (Figure 5A, Equation (3), *p* < 2.2 × 10^−16^, left-tailed paired Wilcoxon signed-rank test). The strains from different clades were clustered together without clear separation (Figure 5B). These results indicated that in addition to mutation pressure, natural selection played an important role in shaping codon usage patterns in MPXV. In addition, a Spearman’s rank correlation analysis revealed a mixture of significant positive and negative correlations between the nucleotide compositions, ENC, Dim.1, and Dim.2 (Figure 6). More specifically, all the composition constraints were remarkably correlated with ENC and Dim.2, with correlation coefficients ranging from −0.88 to +0.94 (*p* < 0.05). These results confirmed that mutation pressure and natural selection jointly shaped codon usage patterns in MPXV.

Furthermore, neutrality plot analyses were conducted to estimate the magnitude of mutation pressure and natural selection in structuring codon usage in MPXV. A significant correlation was observed between the GC12s and GC3s for all strains (R^2^_adj_ = 0.2, *p* < 0.0001), irrespective of the clade (Figure 5C). The slope of the regression line was 0.27, signifying that relative neutrality (mutation pressure) accounted for 27% of the influence, whereas natural selection accounted for 73%. This result indicated that natural selection dominated the codon usage patterns in MPXV. However, although the dominant effect of natural selection was still observed in the WA-Others and CA clades with slope values of 0.18 and 0.22, the WA-Outbreaks exhibited a different trend (Figure 5D). For the strains in the WA-Outbreak clade, a significant correlation between GC12s and GC3s was found (slope 0.72, R^2^_adj_ = 0.56, *p* < 0.0001), and mutation pressure and natural selection were 72% and 28%, respectively, suggesting that mutation pressure predominantly determined the coding sequences of the 2022 outbreak strains over natural selection. 

### 2.7. Distinct Patterns of MPXV Adaptation to Potential Hosts

**Figure 7:**
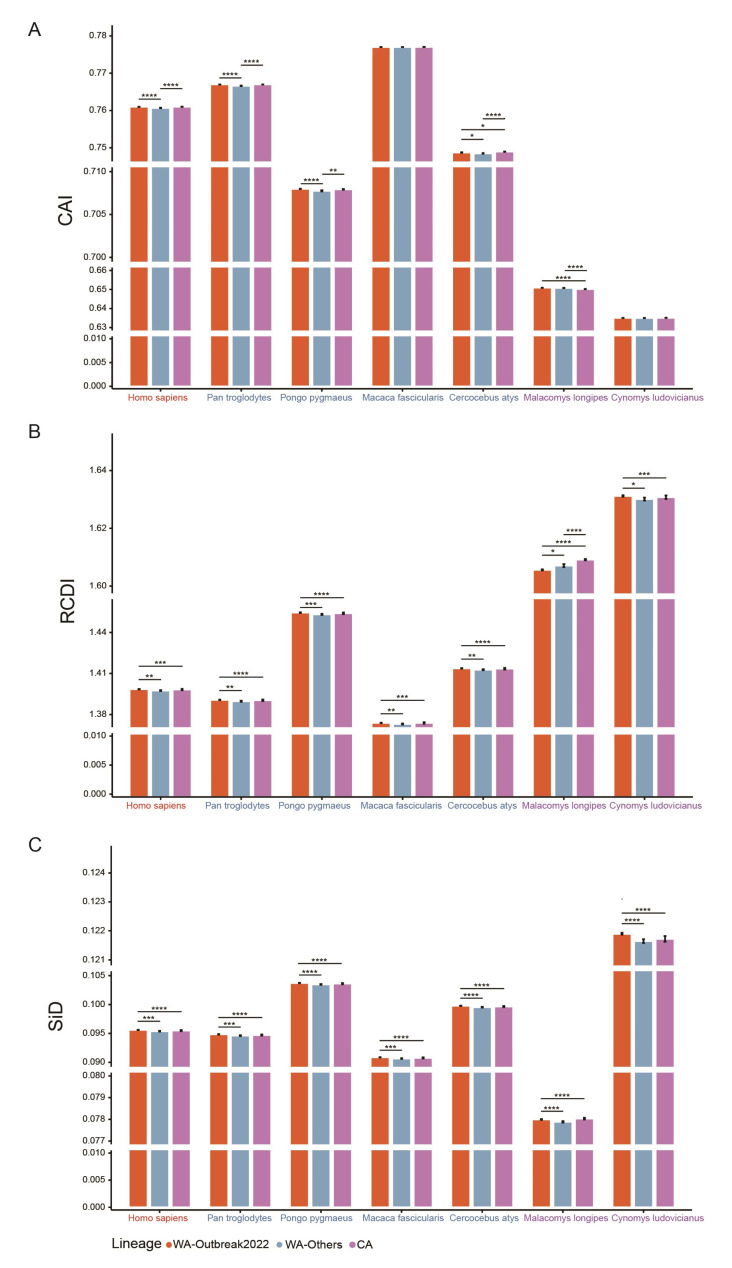
(**A**) CAI, (**B**) RCDI, and (**C**) SiD analyses of codon usage for MPXV and its potential hosts. Benjamini–Hochberg (BH)-corrected Dunn’s test was used for comparisons between groups. All differences with *p* < 0.05 are indicated. * *p* < 0.05; ** *p* < 0.01; *** *p* < 0.001; **** *p* < 0.0001. The colors of labels on the horizontal axis correspond to the reference genome: humans (red), NHP (blue), and rodents (purple).

To investigate the adaptation of the MPXV to its potential hosts, a CAI analysis was performed. The CAI values varied from host to host. The highest CAI values were obtained for *M. fascicularis* (0.7770 ± 0.0001), followed by *P. troglodytes* (0.7669 ± 0.0003), *H. sapiens* (0.7609 ± 0.0003), *C. atys* (0.7487 ± 0.0004), *P. pygmaeus* (0.7079 ± 0.0003), *M. longipes* (0.6507 ± 0.0005), and *C. ludovicianus* (0.6350 ± 0.0003). At the clade level, though the absolute difference did not exceed 0.0004, the CAI values of the WA-Outbreak2022 clade were significantly higher than those of the WA-Others clade for *H. sapiens*, *P. troglodytes*, *P. pygmaeus*, and *C. atys* (adjusted *p* < 0.05, Dunn’s test, Figure 7A), indicating that the 2022 outbreak strains were better adapted to these hosts. The CAI values of the CA clade were also remarkably higher than those of the WA-Others clade for *H. sapiens*, *P. pygmaeus*, and *C. atys*, but lower than those for *M. longipes*.

RCDI was also analyzed to further explore the adaptation of MPXV to various hosts. MPXV displayed the highest RCDI values for *C. ludovicianus* (1.6308 ± 0.0032), followed by *M. longipes* (1.6070 ± 0.0027), *P. pygmaeus* (1.4540 ± 0.0028), *C. atys* (1.4134 ± 0.0026), *H. sapiens* (1.3981 ± 0.0025), *P. troglodytes* (1.3903 ± 0.0025), and *M. fascicularis* (1.3736 ± 0.0024), indicating that the coding sequences of MPXV showed the biggest codon deoptimization to *C. ludovicianus*. At the clade level, the highest and lowest RCDI values were obtained from *C. ludovicianus* and *M. fascicularis*, regardless of the clade (Figure 7B).

### 2.8. Selection Pressure Driven by Hosts on MPXV

To evaluate the influence of potential hosts on the evolution of the codon usage pattern in MPXV, the SiD analysis was performed. Among seven potential hosts, *C. ludovicianus* exhibited the highest SiD values (0.1218 ± 0.0005), followed by *P. pygmaeus* (0.1036 ± 0.0005), *C. atys* (0.0997 ± 0.0005), *H. sapiens* (0.0955 ± 0.0005), *P. troglodytes* (0.0947 ± 0.0005), *M. fascicularis* (0.0908 ± 0.0004), and *M. longipes* (0.0780 ± 0.0002). This result suggested that *C. ludovicianus* had a much stronger impact on the overall codon usage preferences of MPXV than those of the other hosts. A clade-wise SiD analysis showed a similar pattern (Figure 7C). Moreover, the SiD values of the WA-Outbreak2022 were significantly higher than those of the WA-Others and CA for all hosts except *M. fascicularis*, where the SiD values of the WA-Outbreak and CA were comparable but remarkably higher than those of WA-Others (Figure 7C). Accordingly, the codon usage patterns of the WA-Outbreak2022 clade were much more influenced by selection pressure induced by hosts. 

## 3. Discussion

In this study, we comprehensively analyzed the evolution of MPXV 2022 outbreak strains from the perspective of codon usage. According to our ML phylogenetic tree, the MPXV strains responsible for the 2022 worldwide outbreak belonged to the WA lineage (referred to as “WA-Outbreak2022” in this study). PCA based on the RSCU values revealed two separate clusters, which confirmed the phylogenetic relationships and suggested that the codon usage preferences contributed to the evolution of the MPXV. Notably, the 2022 outbreak strains formed two distinct branches in the phylogenetic tree. The most similar pre-2022 outbreak strains in the two branches were isolated from two unrelated cases [20] and had more than 35 unique nucleotide differences, implying that the ongoing outbreak possibly possesses multiple transmission origins. Meanwhile, the two branches of the 2022 outbreak clustered together with the 2017–2018 Nigeria outbreak strains and related exportation cases [4], suggesting that the 2022 outbreaks might be a consequence of the ongoing circulation and evolution of the MPXV that led to the 2017–2018 Nigeria outbreak [22].

The nucleotide compositions of a genome could affect its codon usage. Here, we found that the coding sequences of the MPXV were A/T-rich. Of note, the WA-Outbreak2022 strains had slightly but significantly higher A/T contents, and these may be associated with prevailing GA > AA and TC > TT mutation biases in their genomes [20,22,25]. The RSCU analysis also demonstrated that preferred codons were almost always A- and T-ended, suggesting that nucleotide compositions contributed to the codon usage patterns in MPXV. In addition, the ENC analysis indicated weak overall CUB. Our results agreed with those of a previous report [23]. Similar CUB was also observed in other viruses, such as HTNV (47.52) [26] and PEDV (48.1) [27]. Such low CUB might facilitate their survival and replication within the host via minimizing the protein synthesize resource competition between the host and inner viruses [18,28]. Clade-wise analysis suggested that the WA-Outbreak2022 strains exhibited a slightly but remarkably greater CUB than the strains of WA-Others and CA. In general, higher CUB (i.e., lower ENC) is associated with higher gene expression [28,29]. Given the ever-expanding area of transmission and increasing infection cases [2,30,31], viruses in the WA-Outbreak2022 clade may continue to evolve and mutate in the “evolutionary arena”, resulting in a greater CUB to support their survival and further spread in potential hosts.

Mutation pressure and natural selection are usually considered the main factors influencing codon usage. Our ENC-GC3s plot suggested that natural selection played a role in MPXV codon usage preferences along with mutation pressure. Correlation analysis confirmed that both mutation pressure and natural selection contributed to the observed codon usage patterns. Moreover, neutrality plots suggested that natural selection was the major driver of codon usage preferences in the WA-Others and CA clade, whereas mutation pressure dominantly explained the CUB in the WA-Outbreak2022, indicating that the 2022 monkeypox outbreak strains may undergo a unique evolutionary history, differing from those in the WA-Others and CA clades. This unique evolutionary history was also supported by phylogenetic analysis and mutation characterization [20,32,33].

Successful viral survival and replication require the cellular structure and resources of the host. To further study the interrelationship between MPXV and its potential hosts, detailed CAI, RCDI, and SiD analyses were conducted. Larger CAI values indicate better adaptation, reflecting the role of natural selection [34]. Our CAI analysis results revealed that the MPXV was well-adapted to primates (*M. fasciculari*, *P. troglodytes*, and *H. sapiens*) but not to rodents (*M. longipes*, and *C. ludovicianus*), consistent with the results of the RCDI analysis. These results agreed with the results of epidemiologic monitoring in which MPXV was largely isolated from primates. It has been postulated that a lower level of adaptation to natural reservoirs than to terminal hosts may help the virus maintain a long-term co-existence and circulation within natural reservoirs [18,35]. Therefore, the rodents may be the natural reservoirs of MPXV, as proposed in previous reports [23,36,37]. More importantly, the 2022 outbreak strains were slightly better adapted to many hosts, including humans, compared to other viruses of the WA lineage. The greater adaptation to humans might be related, at least in part, to the multi-country transmission and outbreak, as increasing cases of human-to-human transmission were reported [38,39,40]. However, the levels of adaptation require further investigation, especially in wet experiments. Additionally, the SiD analysis suggested that *C. ludovicianus* exerted much stronger selection pressure on the CUB of the MPXV. The high SiD values also implied *C. ludovicianus* may be a natural reservoir, although this result should be carefully interpreted and further examination is required, particularly because the codon usage table of the *species* was built from 17 CDSs and may be highly biased. Nevertheless, selection induced by hosts clearly played an important role in shaping the codon usage pattern in MPXV, as revealed by the relatively high SiD values for primates such as *P. pygmaeus*, *C. atys*, and *H. sapiens*. This is contrary to previous results showing that natural selection from hosts did not influence the CUB of the MPXV [23]. Furthermore, considering clade information, the SiD analysis demonstrated that 2022 outbreak strains underwent significantly stronger selection pressure induced by the host, regardless of the host. It is possible that the 2022 outbreak strains underwent continuous APOBEC3 deaminase editing in some host(s) during recent evolution [20,22,41]. Compared to ancestral strains from previous outbreaks, most nucleotide mutations in the WA-Outbreak2022 viral genomes were either GA > AA or TC > TT in the dinucleotide context, which is a specific signature of APOBEC3 deaminase editing [41]. The putative APOBEC3 editing effect on WA-Outbreak2022 MPXV may play a part in shaping their biased A- and T-ended preferred codons usage pattern.

In conclusion, we performed a comprehensive analysis of the evolution of MPXV from the codon usage perspective, with an emphasis on the 2022 monkeypox outbreak strains. Our results suggested the overall CUB among MPXVs is not highly biased, and the 2022 outbreak strains show slightly but significantly higher CUB than those of the other strains. Both mutation pressure and natural selection influenced the codon usage patterns of the MPXV; however, mutation pressure dominantly determined the codon usage patterns of the 2022 outbreak strains, differing from the dominant processes shaping the WA-Others and CA lineage. Additionally, we found that the 2022 outbreak strains had significantly higher adaptation to many hosts, such as humans, and underwent stronger selection pressure induced by hosts. Our findings are expected to improve our understanding of the evolutionary biology of the MPXV and its host adaptation.

## 4. Materials and Methods

### 4.1. Dataset

All publicly available full-length genome sequences (≥177,739 bp, 90% of the reference size) and coding sequence annotations of the MPXV isolates were downloaded from the GenBank database on 13 June 2022. For strains without CDS annotation in GenBank, genome annotation was acquired using VAPiD [42]. The sequences were filtered to retain high-quality genomes as follows: (a) the content of N bases less than 1.5%; (b) degenerate base content less than 5%; (c) length of the CDS region ≥ 100 bp; (d) the proportion of non-deterministic bases (N and degenerate bases) in coding sequences ≤ 1.5%; and (e) the CDS must be able to be properly translated. The screened CDSs were concatenated to generate a complete coding sequence for each genome. The genomes whose concatenated coding sequences were < 160 kb were removed. A total of 161 high-quality genomes (69 strains from the 2022 outbreak, 46 strains classified as WA-Others, and 46 strains in the CA clade) and their complete coding sequences were retained for subsequent analysis. Detailed information on these strains, including the accession number, isolated host, country, and collection date, is listed in Table S1. 

### 4.2. Recombination Analysis

To exclude the effects of recombination events on the CUB of MPXV, seven methods for recombination detection were implemented in RDP5 (Recombination Detection Program version 5) [43], including RDP, Chimaera, SiScan, GENECONV, Bootscan, MaxChi, and 3 Seq. As described previously [44], a recombination event was considered a true positive when at least six of the seven methods yielded significant *p*-values. No valid recombination event was identified and, hence, all of the genomes were included in further analyses.

### 4.3. Phylogenetic Analysis

The whole genomes were aligned using MAFFT v7.505 [45]. The alignment was applied to IQ-TREE v2.1.4 to build a maximum likelihood (ML) phylogenetic tree [46]. The tree topology was evaluated using 1000 replications of ultrafast bootstrap resampling [47] and the SH-aLRT test [48]. The bit-fit nucleotide substitution model K3Pu+F+R8 was selected using ModelFinder [49]. The phylogenetic tree was visualized using the ggtree package [50]. 

### 4.4. Codon Usage Bias Analysis

#### 4.4.1. Nucleotide Composition Analysis

The CUB analysis was performed as described in our previous paper [51]. In brief, the abundance of the mononucleotides (A, C, G and T) and GC contents at the first (GC1s), second (GC2s), and third (GC3s) codon positions were calculated using the seqinr package in R [52]. The frequencies of nucleotides (A, C, G, T) at the third positions in synonymous codons (A3s, C3s, G3s, and T3s) were inferred using program CodonW [53]. Mean values of GC1s and GC2s (GC12s) were also computed. 

#### 4.4.2. The Effective Number of Codons (ENC) Analysis

The ENC value, which ranges from 20 to 61, shows the degree of CUB. The stronger CUB is indicated by a lower ENC value. The seqinr package was used to calculate the ENC values using the following formula [29]:

(1) $$\;ENC=2+\frac{9}{F_{2}^{\prime }}+\frac{1}{F_{3}^{\prime }}+\frac{5}{F_{4}^{\prime }}+\frac{3}{F_{6}^{\prime }}\;\;\;\;\;\;$$

where $F_{k}^{\prime }$ (k = 2, 3, 4, or 6) refers to the mean value of homozygosity (*F_k_*) for the k-fold degenerate amino acids. The *F_k_* value was estimated using the following formula:

(2) $$F_{k}=\frac{n\cdot \sum_{i=1}^{k}{\left(\frac{n_{i}}{n}\right)}^{2}-1}{n-1}$$

where *n* is the total number of occurrences of the codon for that amino acid; *n_i_* is the total number of observed *i*-th codon for that amino acid. Genes whose codon usage is only limited by mutation pressure will fall on or near the curve of the expected ENC values. Otherwise, natural selection makes a stronger impact. Hence the ENC-plot analysis was carried out by plotting the ENC values against the GC3s in order to determine the factors affecting CUB. The formula as follows was used to determine the expected ENC value [29]:

(3) $${ENC}_{expected}=2+s+\frac{29}{s^{2}+(1-{s)}^{2}}$$

where the *s* represents values of GC3s. The ENC-plot was conducted by using R script with ggplot2 package [54].

#### 4.4.3. The Relative Synonymous Codon Usage (RSCU) Analysis

The RSCU values represent the ratio of the observed value to the expected value of the specific codon in the synonymous codon family, given that all codons for the particular amino acid are used equally. It excludes the effect of the sequence length and amino acid compositions. The RSCU value was calculated using the seqinr package as follows [52,55]:

(4) $$RSCU=\frac{X_{ij}}{\frac{1}{N_{i}}\sum_{j}^{N_{i}}X_{ij}}\;\;\;\;\;$$

where X*_ij_* is the observed number of *j*-th codon for the *i*-th amino acid, which has *N_i_* kinds of alternative synonymous codons. Codons with RSCU values greater than 1.0 indicate a positive bias in codon usage and are referred to as “abundant” codons. Conversely, those with RSCU values less than 1.0 suggest a negative bias in codon usage and are considered “less-abundant” codons. If the RSCU value is exactly 1.0, there is no bias in codon usage for that particular amino acid, and its selection of codons is either random or equal. Typically, codons with RSCU values > 1.6 are considered as over-represented, whereas <0.6 reflected under-represented ones.

#### 4.4.4. Principal Component Analysis (PCA)

PCA is a commonly used method for exploring the relationships between multivariate data and samples. In this study, a 59-dimensional vector was used to represent each sequence, where each dimension represents an RSCU value of a synonymous codon. The matrix consisted of 59 RSCU values per virus and was transformed into several principal components (PCs) using PCA. The analysis was carried out using the factoextra package [56], which provides a range of functions for visualizing and extracting results from multivariate data analyses, including PCA.

#### 4.4.5. Neutrality Plot Analysis

The neutrality plot was performed to investigate the magnitude of influences of natural selection and mutation pressure on the CUB of the MPXV by plotting the GC12s values (y-axis) against the GC3s values (x-axis) [16]. It has been proposed that the slope of the plot reflects the extent of influences of mutation pressure. If the slope is statistically significant and close to 1, mutation pressure is thought to be the dominant factor influencing codon usage. A slope value that is closer to 0 indicates that natural selection has a greater influence. This analysis was conducted using R script and the ggplot2 package [54].

#### 4.4.6. Codon Adaption Index (CAI) Estimation

The CAI is a simple but effectively quantitative approach to measuring the relative adaptation of a gene towards codons of highly expressed genes [34]. The CAI values of MPXV in relation to the synonymous codon usage patterns of its potential hosts were estimated using the standalone CAIcal software (v1.4) [57]. Seven reference datasets of synonymous codon usage patterns of the three classes of potential hosts, including humans (*Homo sapiens*), non-human primates (NHP, *Pan troglodytes*, *Pongo pygmaeu, Macaca fascicularis*, and *Cercocebus atys*), and rodents (*Malacomys longipes* and *Cynomys ludovicianus* (one species of prairie dog)), were downloaded from the Codon and Codon-Pair Usage Tables (CoCoPUTs) database on 23 June 2022 [58]. The higher CAI values, ranging from 0 to 1, reflect better relative adaptation to the related potential hosts.

#### 4.4.7. Relative Codon Deoptimization Index (RCDI) Computation

The RCDI value evaluates how much the MPXV has deoptimized in comparison to its hosts. An RCDI value of 1 indicates that the codon usage patterns are similar and exhibits a host-adapted codon usage preference, while RCDI values higher than 1 suggest the codon usage pattern of the virus deviates from its host and thus has lower adaptability [59]. The local version of CAIcal was employed to compute RCDI values [57].

#### 4.4.8. Similarity Index Analysis

The similarity index (SiD or D(A, B)) is an indicator to measure the overall influence of host codon usage patterns on viral codon usage. The SiD values were calculated using the following equation by R script [60]:

(5) $$R\left(A,B\right)=\frac{\sum_{i=1}^{59}a_{i}\cdot b_{i}}{\sqrt{\sum_{i=1}^{59}{a_{i}}^{2}\cdot \sum_{i=1}^{59}{b_{i}}^{2}}}\;\;\;\;\;\;\;\;\;$$

(6) $$D\left(A,B\right)=\frac{1-R(A,B)}{2}\;\;\;\;\;\;\;\;\;\;\;\;\;\;$$

where *a_i_* and *b_i_* represent the RSCU value of the *i*-th codon among the 59 synonymous codons for the MPXV and its host, respectively. D(A, B) represents the potential influence of the overall codon usage patterns of the host on that of USUV, ranging from 0 to 1. Higher SiD values are considered to imply a greater impact from the host on the codon usage pattern of MPXV.

#### 4.4.9. Correlation and Statistics Analysis

Spearman’s rank correlation analysis was conducted to evaluate the relationships among the nucleotide compositions, ENC, and the first two axes of the PCA. The results were visualized using the R packages ggplot2, ggbreak, ggpubr, and ggcorrplot [54,61,62,63]. To determine the statistical significance between groups, a two-sided Dunn’s test was employed. The Benjamini–Hochberg (BH) procedure was used to correct *p* values, and 0.05 was chosen as the significance threshold.

## Data Availability

The data used in this study are available in GenBank. A full of accession numbers of genomes are listed in Table S1.

## Supplementary Materials

The following supporting information can be downloaded at: https://www.mdpi.com/article/10.3390/ijms241411524/s1.
